# Implementation science in maternity care: a scoping review

**DOI:** 10.1186/s13012-021-01083-6

**Published:** 2021-02-04

**Authors:** Ann Dadich, Annika Piper, Dominiek Coates

**Affiliations:** 1 Western Sydney University, School of Business, Locked Bag 1797, Penrith, NSW 2751 Australia; 2 University of Technology Sydney, Broadway, PO Box 123, Ultimo, NSW 2007 Australia

**Keywords:** Maternity care, Pregnancy, Childbirth, Scoping review, Knowledge translation, Theory, Model, Framework

## Abstract

**Background:**

Despite wide recognition that clinical care should be informed by the best available evidence, this does not always occur. Despite a myriad of theories, models and frameworks to promote evidence-based population health, there is still a long way to go, particularly in maternity care. The aim of this study is to appraise the scientific study of methods to promote the systematic uptake of evidence-based interventions in maternity care. This is achieved by clarifying if and how implementation science theories, models, and frameworks are used.

**Methods:**

To map relevant literature, a scoping review was conducted of articles published between January 2005 and December 2019, guided by Peters and colleagues’ (2015) approach. Specifically, the following academic databases were systematically searched to identify publications that presented findings on implementation science or the implementation process (rather than just the intervention effect): Business Source Complete; CINAHL Plus with Full Text; Health Business Elite; Health Source: Nursing/Academic Edition; Medline; PsycARTICLES; PsycINFO; and PubMed. Information about each study was extracted using a purposely designed data extraction form.

**Results:**

Of the 1181 publications identified, 158 were included in this review. Most of these reported on factors that enabled implementation, including knowledge, training, service provider motivation, effective multilevel coordination, leadership and effective communication—yet there was limited expressed use of a theory, model or framework to guide implementation. Of the 158 publications, 144 solely reported on factors that helped and/or hindered implementation, while only 14 reported the use of a theory, model and/or framework. When a theory, model or framework was used, it typically guided data analysis or, to a lesser extent, the development of data collection tools—rather than for instance, the design of the study.

**Conclusion:**

Given that models and frameworks can help to describe phenomenon, and theories can help to both describe and explain it, evidence-based maternity care might be promoted via the greater expressed use of these to ultimately inform implementation science. Specifically, advancing evidence-based maternity care, worldwide, will require the academic community to make greater explicit and judicious use of theories, models, and frameworks.

**Registration:**

Registered with the Joanna Briggs Institute (registration number not provided).

**Supplementary Information:**

The online version contains supplementary material available at 10.1186/s13012-021-01083-6.

Contributions to the literature

- Aligning healthcare with evidence-based practice can be challenging—what clinicians do, how they do it, when they do it, and who they do it with, is shaped by myriad factors and processes.
- Implementation science in maternity care was helped or hindered by: organisational factors (culture, communication, coordination, stakeholder engagement and implementation planning); personal factors (motivation, perceived value, knowledge and skill development) and contextual factors (adaptation of the intervention and/or its implementation, the capacity to accommodate change and infrastructure).
- Although theory can clarify how different practices are introduced, operationalised and sustained, only 6 of 158 publications explicitly referred to a theory.

## Background

Despite wide recognition that clinical care should be informed by the best available evidence, this does not always occur [1, 2]. Internationally, policymakers, health service managers, clinicians and scholars struggle to promote evidence-based practice [3]. Although evidence-based (or -informed) clinical guidelines are produced at an increasing rate, they are not routinely translated into clinical care [4].

Changing the ways that healthcare is delivered, managed or experienced can be difficult [5]. This is because healthcare is shaped by myriad factors and processes—be they personal, social, organisational, economic or institutional [4, 6, 7]. Merely relying on clinicians to make sense of, and adapt the information presented in written artefacts, like refereed journals and clinical guidelines, is (highly) unlikely to promote evidence-based (or -informed) healthcare [8, 9]. A linear understanding of evidence translation—from ‘bench to bedside’ [10]—is naïve. This is because those who deliver, manage and receive healthcare, negotiate multiple forms and sources of evidence, which complement and compete with each other [2, 11, 12] within a complex system of institutional logics [11, 13].

To advance evidence-based population health, implementation science has emerged to ‘promot[e]… the uptake of research findings into healthcare practice and health policy’ [14]. Specifically, it represents:

the scientific study of methods to promote the systematic uptake of evidence-based interventions into practice and policy and hence improve health. In this context, it includes the study of influences on professional, patient and organisational behaviour in healthcare, community or population contexts.

Informing (and from) these scientific pursuits are theories, models and frameworks [15]. According to Nilsen [16], these can be categorised by their expressed aim. Although interrelated, there are those that (largely) ‘describ[e]… and/or guid[e]… the process of translating research into practice’; there are those that (largely) aim to ‘understand… and/or explain… what influences implementation outcomes’; and there are those that (largely) ‘evaluat[e]… implementation’. Guided by these aims, Nilsen helpfully developed a taxonomy comprised of five categories—reflecting his order, these include process models, like that of Landry and colleagues [17]; determinant frameworks, like that of Damschroder and colleagues [18]; classic theories, like social cognitive theories [19]; implementation theories, like the normalisation process theory [20]; and evaluation frameworks, like the oft-cited RE-AIM [21] and PRECEDE-PROCEED [22]. Despite the myriad theories, models and frameworks to promote evidence-based population health, there is still a long way to go [23, 24], particularly in maternity care [25–28].

There is a limited understanding of the evidence that is (and is not) translated into maternity care, the associated reasons and how population health can be bolstered via evidence-based maternity care [25]. This warrants concern for (at least) three key reasons. First, quality maternity care is ‘fundamental to good public health’ [29]. Spanning the care of ‘women during pregnancy, childbirth and the postnatal period’ [30], maternity care can bolster the foundation required for healthy development, from infancy to adulthood. Second (and relatedly), it can serve to prevent health and/or mental health issues, or at least open opportunities for early intervention. Third, quality maternity care can help to address longstanding health inequities that compromise population health in low- and middle-income countries. As the World Health Organization attested:

About *810* women *die* from pregnancy- or childbirth-related complications *every day*. *94%* of all maternal deaths occur in *low* and *lower middle-income* countries ([31], emphasis added).

*Sub-Saharan Africa* and *Southern Asia* accounted for approximately *86%* (254 000) of the estimated global *maternal deaths* in 2017. Sub-Saharan Africa alone accounted for roughly two-thirds (196 000) of maternal deaths, while Southern Asia accounted for nearly one-fifth (58 000)… [However] Most maternal deaths are *preventable*, as the health-care solutions to prevent or manage complications are well known ([32], emphasis added).

It is perhaps for these (and other) reasons that maternal health is one of eight United Nations millennium development goals [33].

Given the key role of maternity care in evidence-based population health, the aim of this study is to appraise the scientific study of methods to promote the systematic uptake of evidence-based interventions in maternity care by clarifying if and how implementation science theories, models and frameworks are used. This was achieved via a scoping review of publications, identified via a systematic search of academic databases, to ultimately ‘map the existing literature in a field of interest in terms of the volume, nature and characteristics of the primary research’ [34]. Relative to other approaches—like a systematic review or meta-analysis—a scoping review was deemed appropriate for two key reasons. First, given the absence of a systematic review in this area, a scoping review can ‘inform a systematic review, particularly one with a very broad topic scope’, like implementation science in maternity care [35]. Second, scoping reviews are ‘the better choice’ [36] when ‘identif[ying]… certain characteristics/concepts in papers or studies, and… mapping, reporting or discussi[ng]… these characteristics/concepts’. Given these reasons, a scoping review was conducted, guided by Peters and colleagues’ [37] approach. This involved ‘at least two reviewers’; ‘an a priori scoping review protocol’; ‘predefine[d]… objectives and methods… and details the proposed plans’; and—‘due to the more iterative nature of a scoping review’—‘changes [were]… detailed and justified… if and when they occur’.

## Methods

### Searches

A protocol was developed, as per the preferred reporting items for systematic reviews and meta-analyses extension for scoping reviews (PRISMA-ScR; see Additional file 1) [38]. This protocol specified: the population of interest—namely, maternity care settings, irrespective of geographical location; the phenomenon of interest—namely, the use of implementation science in maternity care; as well as the outcomes—namely, the theories, models and frameworks used to inform the research; the associated effects; and the factors that helped or hindered the implementation. As a scoping review of implementation science in maternity care, presented in narrative form, there was no intervention or comparator—as such, these components of the protocol were not applicable. To the authors’ knowledge, no similar review had been published or was in development. This was ascertained by searching academic databases and the online platforms of organisations that register review protocols—namely, PROSPERO and the Joanna Briggs Institute. The protocol was therefore registered with the Joanna Briggs Institute (registration number not provided). Given their relevance to the study aim, the following academic databases were systematically searched to identify relevant refereed publications: Business Source Complete; CINAHL Plus with Full Text; Health Business Elite; Health Source: Nursing/Academic Edition; Medline; PsycARTICLES; PsycINFO; and PubMed. Grey literature was purposely excluded to optimise the veracity of the findings. The academic databases were searched in December 2019 by searching for the following terms within publication title and/or abstract: ‘implementation’ and ‘maternity’. This approach was used because, after testing variations—for instance, a search of keywords or the full-text, including references—this strategy helped to ensure focus and comprehensiveness.

### Inclusion criteria

A publication was included in this review if it presented findings on implementation science or the implementation process (rather than simply the effect of an intervention), as per the study focus, irrespective of study design; represented a research publication (rather than a letter, commentary, protocol or an editorial) to ensure the inclusion of empirical research; was authored by a named (rather than an anonymous) author, to exclude non-empirical research; was published in English, irrespective of the geographical location of the study site(s), to ensure the authors could directly review each publication, while ensuring no geographical location was excluded; was published from 2005 onwards (inclusive) to optimise the currency and potential relevance of key findings; and/or did not represent a systematic, narrative or literature review or meta-analysis, given the limited detail typically reported from the publications that are included within such reviews. To optimise robustness, AD, AP and DC independently reviewed 100 publications, and all authors discussed and reconciled differences. Following this, AP vetted the title and abstract of the remaining publications and analysed the full text of those that remained. All authors determined the publications that warranted discussion, following due consideration of the full text.

### Data extraction, data synthesis and study quality assessment

Once irrelevant publications were excluded, the remaining were analysed. Specifically, using Microsoft Word and Excel, AP extracted content regarding: publication details (namely, the title, author, year, nation, population, aim, context and methods); the use of a theory, model and/or framework to guide implementation, as per Nilsen’s [16] categories—namely, classic theories, determinant frameworks, implementation theories, evaluation frameworks and process models; the factors that helped or hindered implementation; key findings; as well as author-identified limitations and future research opportunities. The Excel-based extraction tool was used with the first ten publication and was deemed to be fit-for-purpose. Following this, AP tabulated the aforesaid content from the remaining publications and reported on key findings in narrative form. The publications included in this review contained sufficient detail on the methods used to promote the systematic uptake of evidence-based interventions in maternity care—as such, the authors of these publications were not contacted for further information or their data. Because this review purposely focused on implementation science in maternity care (as opposed to the effects associated with an intervention), a systematic assessment of study quality was not conducted. Furthermore, because the publications were refereed, their content was assumed to be accurate and valid.

## Results

### Review statistics

**Fig. 1:**
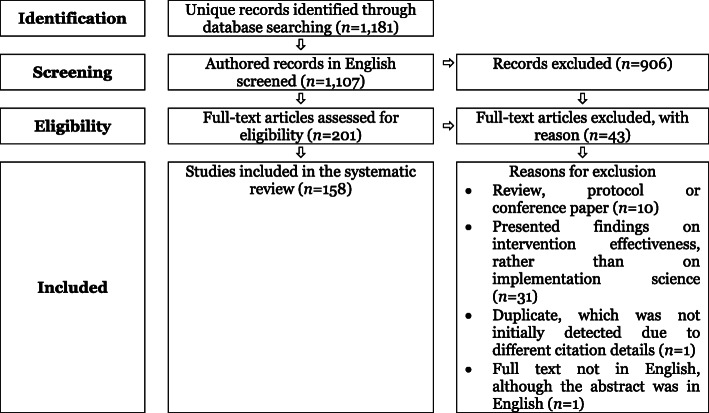
Flow diagram of publication selection (adaption of PRISMA; [39])

Of the 1181 unique publications initially identified, 158 were included in this review (see Fig. 1). Of these, 144 solely reported the factors that helped and/or hindered implementation (91.1%; see Table 1), while only 14 reported the use of a theory, model and/or framework (8.9%).

**Table 1:** Study characteristics (*n* = 144)

Characteristic	*N*°	Publications
Study design		
Qualitative		
Cross-sectional survey	72	[40–111]
Mixed-methods	27	[112–138]
Case study	12	[139–150]
Pre-post study	8	[151–158]
Ethnography	6	[159–164]
Cohort study	4	[165–168]
Pilot-test	8	[169–176]
Longitudinal survey	2	[177, 178]
Quasi-experimental	1	[179]
Randomised controlled trial	2	[180, 181]
Retrospective medical record and document analysis	2	[182, 183]
Region		
Africa	42	[41–43, 45, 47, 49–53, 56, 60, 79, 82, 92, 103, 111, 115–118, 121, 122, 132, 139–142, 148, 151, 152, 159, 160, 169, 170, 173, 174, 176–178, 181, 182]
Europe	36	[58, 61, 66, 69, 72, 76, 81, 83, 85, 87, 88, 91, 97, 98, 100–102, 104, 105, 128, 129, 131, 134–138, 145, 146, 150, 153, 158, 162, 163, 167, 171]
Australia and/or New Zealand	26	[54, 62, 67, 70, 73, 77, 78, 80, 84, 86, 89, 90, 94, 95, 108–110, 120, 123, 125, 126, 143, 144, 154, 157, 175]
United States and/or Canada	14	[68, 71, 74, 96, 99, 107, 119, 130, 147, 149, 156, 172, 179, 183]
Asia	13	[46, 48, 55, 63, 93, 113, 133, 155, 161, 165, 166, 168, 180]
Multiple continents	7	[40, 75, 106, 112, 114, 127, 184]
South and Central America	6	[44, 57, 59, 64, 65, 164]
National income level		
High	83	[54, 58, 61–78, 80, 81, 83–91, 94–102, 104–110, 119, 120, 123, 125, 126, 128–131, 134–138, 143–147, 149, 150, 153, 154, 156–158, 162, 163, 167, 168, 171, 172, 175, 179, 183, 184]
Lower-middle	24	[42, 43, 48–50, 53, 79, 82, 93, 111, 113, 115, 117, 118, 132, 139, 140, 142, 151, 170, 174, 177, 178, 180, 182]
Upper-middle	17	[44, 55–57, 59, 60, 92, 103, 121, 122, 133, 152, 155, 164, 166, 173, 176]
Low	16	[41, 45–47, 51, 52, 114, 116, 141, 148, 159–161, 165, 169]
Multiple nations with a high-, low- and middle-income classification	2	[40, 112]
Multiple nations with a low- and lower-middle-income classification	2	[127, 181]
Participants		
Maternity care clinicians and/or pregnant women	129	[40, 41, 43, 45–48, 50, 51, 53–72, 74–101, 104–112, 114–117, 119–123, 125–131, 133–140, 143–146, 148–170, 172–179, 181, 182, 184]
Parents, health administrators, policymakers, project staff, maternity care clinicians, community outreach workers, and/or community members	10	[42, 44, 52, 73, 103, 113, 118, 132, 147, 171, 180]
Policymakers	3	[49, 141, 142]
Nil—secondary data sourced from case-notes, patient records, and/or guidelines	2	[102, 183]
Context		
Hospital wards	107	[40, 42, 44–46, 48–60, 62–71, 74–86, 88–95, 98–100, 102–109, 112, 114, 115, 117, 120, 122, 125, 128–130, 132–134, 138, 140, 144–147, 149, 151, 152, 154, 155, 157–164, 166–170, 172–177, 181–184]
Community and hospital	24	[41, 47, 61, 87, 97, 101, 110, 116, 118, 123, 126, 127, 131, 137, 139, 141–143, 153, 165, 171, 178–180]
Community	12	[43, 72, 73, 96, 111, 113, 119, 121, 136, 148, 150, 156]
General practices	1	[135]
Research methods		
Mixed-methods	53	[44, 45, 50, 51, 53, 65, 71, 72, 76, 92, 98, 112–115, 119–121, 123, 126, 127, 130–135, 143–145, 148–157, 159, 161–165, 167, 174, 177–179, 181, 184]
Questionnaire or survey	35	[47, 54–56, 61, 63, 67–69, 75, 91, 93–97, 99–109, 111, 116, 136, 137, 158, 166, 168, 173]
Interviews	31	[42, 43, 49, 52, 57, 59, 66, 74, 78, 80–85, 89, 90, 110, 117, 122, 128, 129, 138–140, 142, 146, 171, 172, 175, 176]
Interviews and Focus groups	10	[41, 60, 62, 70, 73, 79, 86, 87, 118, 147]
Focus groups	9	[46, 48, 58, 64, 77, 88, 125, 170, 180]
Case study	4	[40, 141, 182, 183]
Observation	2	[160, 169]
Focus		
Stakeholder perceptions and attitudes re implementation, and/or the associated helpers and hindrances		
Qualitative study	64	[43, 44, 46, 48, 49, 52, 56–60, 62, 66, 69–90, 117, 118, 122, 128–130, 139–143, 145–148, 156, 159–164, 166, 167, 170, 175, 176, 180, 183]
Quantitative and qualitative study	25	[41, 42, 45, 50, 53, 61, 64, 65, 92, 94, 102, 110, 113, 115, 120, 123, 131, 132, 135, 137, 149, 150, 165, 168, 177]
Quantitative study	20	[40, 47, 55, 63, 91, 93, 95–97, 99, 101, 103–109, 111, 152]
Create an implementation theory, model, and/or framework		
Qualitative study	1	[112]
Feasibility testing and/or assess organisational readiness		
Qualitative study	6	[119, 125, 126, 171, 172, 184]
Quantitative and qualitative study	6	[116, 127, 153, 173, 178, 182]
Quantitative study	1	[67]
Use evidence on helpers and hindrances to guide implementation of an intervention		
Quantitative and qualitative study	3	[54, 136, 138]
Qualitative study	2	[68, 155]
Implement and/or pilot–test an intervention		
Quantitative and qualitative study	10	[98, 100, 121, 133, 134, 151, 157, 158, 169, 179]
Qualitative study	5	[51, 114, 144, 154, 174]
Quantitative study	1	[181]
Author-identified limitations		
Methodological issues, including: small sample; recall bias; self-report reliance; and/or limited generalisability	107	[40, 41, 45–49, 52, 54–57, 59–61, 63–66, 68, 70–97, 99–101, 104, 106–113, 115, 119–123, 125, 126, 128, 130, 131, 133–135, 137, 139–141, 143, 145–147, 149–155, 157, 158, 160–163, 165, 167, 168, 170, 171, 173, 176, 178–181, 183]
Nil noted	37	[42–44, 50, 51, 53, 58, 62, 67, 69, 98, 102, 103, 105, 114, 116–118, 127, 129, 132, 136, 138, 142, 144, 148, 156, 159, 164, 166, 169, 172, 174, 175, 177, 182, 184]

### Theories, models and frameworks: absent

The 144 publications that reported on factors that helped and/or hindered implementation noted: organisational factors, including organisational culture, communication, coordination, stakeholder engagement and implementation planning; personal factors, including motivation, perceived value, knowledge and skill development; as well as contextual factors, including the adaptation of the intervention and/or its implementation, the capacity to accommodate change and infrastructure (see Table 2).

**Table 2:** Factors that influenced implementation (*n* = 144)

Factor	Demonstrations	Publications suggesting it helps when present	Publications suggesting it hinders when absent
Organisational	Healthy organisational culture, including: limited tension between disciplines/professions; clearly defined professional roles and responsibilities; interprofessional respect; limited tension between traditional and western medicine; and limited cultural taboos, social stigma, and discrimination against service users	[46, 131]	[40, 42–44, 46, 50, 54, 55, 57, 59, 60, 64–66, 68, 70, 74, 76, 78, 81, 83, 85, 86, 88, 89, 99, 105, 108, 114, 115, 117, 120, 128, 131, 140, 142, 143, 145, 147–149, 155, 160–163, 169, 170, 179, 180, 184]
	Effective communication between and among managers, multidisciplinary service providers, and service users	[46, 48, 51, 52, 54, 56, 60, 66, 71, 75, 78, 80, 88, 91, 92, 98, 101, 102, 105–107, 110, 114, 125, 128, 131–133, 136, 140, 143, 146, 149, 150, 154, 158, 163, 166–168, 172, 177, 179]	[40, 43, 46, 51, 55–57, 59, 60, 64, 68, 73, 76, 85, 86, 89, 94, 97, 114, 115, 119, 122, 123, 125, 126, 132, 141, 142, 149, 159, 162, 171, 174, 179, 182]
	Effective multilevel coordination, support, management, and/or leadership	[40, 41, 44, 46, 48, 50, 52, 56, 57, 62, 66, 75, 79, 84, 86, 89, 94, 98, 99, 101, 103, 106, 110, 113, 114, 119, 123, 125, 131–133, 139, 140, 143, 145, 147, 158, 160, 162–164, 168, 169, 172, 175, 177, 179, 182]	[40, 42–44, 46, 49–51, 55–57, 59, 60, 65, 66, 68, 70, 73, 76, 85, 86, 89, 97, 114, 115, 117–119, 122, 128, 130, 132, 141, 142, 146, 149, 156, 159, 162, 164, 166, 171, 174, 177, 179, 180, 182]
	Stakeholder engagement, including: community engagement; rapport building; local leadership; community awareness initiatives; welcoming community comment; service user involvement in care; and interorganisational networking	[40, 41, 44, 46, 69, 74, 84, 92, 99, 110, 125, 131, 133, 139, 140, 143, 147, 174, 176, 179, 185]	[40, 43, 49, 110, 118, 141, 159, 169, 170, 178]
	Service provider involvement in the design, development, or use of an intervention, and implementation strategy, the evaluation of the intervention, and/or the dissemination of information about the project	[40, 46, 49, 74, 98, 99, 110, 113, 119, 133, 134, 136, 138, 140, 154, 155, 159, 172, 174]	[40, 43, 46, 64, 178]
	Implementation planning, including its stages, pilot–testing, evaluation, and/or sustainability	[40, 46, 79, 84, 92, 98, 114, 119, 123, 125, 140, 143, 146, 147, 153, 172, 174, 175, 183]	[40, 45, 61, 66, 73, 94, 102, 111, 118, 139, 142, 143, 152, 162, 165]
Personal	Motivation to change among service providers	[44, 53, 58, 62, 72, 75, 79, 83, 86, 89, 94, 118, 119, 123, 129, 131, 140, 145, 148, 153, 154, 161, 163, 164, 171, 173, 174, 177, 184]	[40, 42, 43, 48, 49, 51, 53, 56, 58, 59, 61, 63, 68, 70–72, 74, 76, 78, 81, 85, 90, 91, 104, 113–115, 123, 128, 135, 152, 157, 159, 164, 166, 178, 179]
	Perceived value of the intervention among service providers	[47, 55, 56, 58, 63, 70, 72, 76, 78, 80–82, 86, 87, 90, 93, 106, 108, 116, 118, 119, 123, 129, 134, 139, 140, 142, 148, 149, 154, 159, 161, 162, 164, 168, 171, 173, 176, 177, 179, 184]	[40, 48, 49, 56, 59, 113, 123]
	Knowledge, training, education, and/or feedback to or from service providers or service users	[44, 46, 48, 52–54, 56, 61, 63, 71, 79, 81, 84, 86, 88, 91–94, 105, 106, 109, 111, 114, 119, 120, 123, 129, 131, 134, 136, 139–141, 143, 146, 149, 152, 154, 157, 158, 163, 170, 174, 175, 177, 178, 181, 183]	[41, 42, 46, 50–52, 54, 55, 58–60, 63–66, 68, 70, 73, 76–78, 80–82, 86, 87, 90, 97, 99, 107, 109, 119, 121, 122, 130, 135, 139, 141, 147, 148, 157, 161, 165, 166, 168, 171, 177, 178, 180, 186]
Contextual	Adaptation of the intervention and/or its implementation	[46, 76, 84, 94, 96, 97, 103, 110, 119, 125, 131, 140, 145, 153, 164, 171, 172, 174–176, 183]	[40, 49, 50, 63, 131, 141, 142, 159, 169]
	Individual capacity to accommodate change, including: resources; time; working arrangements that align with personal needs; pay incentives to upskill or implement different care models of care; reasonable travel times; and individual wellbeing and work–life balance	[56–58, 61, 62, 84, 111, 118, 120, 140, 164, 172, 177]	[49, 53–55, 60, 61, 63, 90, 97, 108–110, 115, 121, 122, 152, 160, 171–173, 177, 178, 184]
	Organisational capacity to accommodate change, including: workforce capacity; and resources (e.g. medical equipment, administrative equipment, health education materials, time)	[41, 52, 57, 59, 84, 94, 117–119, 140, 141, 143, 152]	[41, 42, 44–52, 54–63, 113, 115–123, 139, 140, 142, 143, 152, 166, 170–173, 177, 180, 182]
	Infrastructure, including: transport; technology; structurally safe and accessible services; adequate physical space in buildings and wards; and reliable water and electricity	[45, 52, 141]	[44–46, 48, 53–55, 58, 60, 63–66, 117, 122, 124, 139, 143, 151, 165, 170, 177, 180]

Of the 144 publications, 58 reported on studies conducted in nations with a low- and/or lower-middle-income (40.3%), as defined by the World Bank [187]—these include two publications that reported on studies conducted across multiple nations with high-, middle- or low-income classifications [40, 112]. Of the 58 publications, 44 cited factors that helped and/or hindered implementation (75.9%)—these included cultural divides, like differences between western and traditional healthcare; the capacity to accommodate change; and infrastructure, particularly limited workforce capacity and resources. These findings highlight the resource implications associated with implementation science in maternity care. Specifically, these publications cited language and cultural barriers that required attention, including norms, fears, tension between western and traditional approaches and stigma [41–43, 113, 114, 139–142, 159–161, 169, 180]; as well as poor patient treatment by staff [115, 139, 170, 180]. In contrast, only one publication re a study conducted in a nation with an upper-middle-income cited tension between western and traditional healthcare as an implementation barrier [44]. Instead, most publications re a study conducted in a more affluent nation spoke of organisational barriers, including: interprofessional tension; poorly defined professional roles and responsibilities; and limited professional autonomy. Collectively, these findings demonstrate the challenges of implementation science in maternity care within nations that are less than affluent. To manage sociocultural barriers, it can be helpful to adapt an intervention to a given context [113, 140, 159, 161]—this might involve engaging with community leaders [42, 43, 139] and/or community members [43, 113, 139–141, 161, 170, 180]. Many of the publications that reported on a study conducted in a lower-middle- or low-income nation also spoke of tangible constraints, including inadequate technology, facilities, transport to these facilities, as well as resources and equipment (59.1%) [41, 42, 45–53, 113, 115–118, 139–142, 151, 165, 170, 177, 180, 182]. In contrast, fewer publications that reported on a study conducted in a high- and/or upper-middle-income nation cited tangible constraints as an implementation barrier (26.0%) [44, 54–66, 119–123, 143, 152, 166, 171–173, 184]. Instead, many spoke of limited space within hospital wards [58, 60, 63–66, 122] or the remoteness of rural maternity services (9.0%) [54, 143].

Some of the 144 publications focused on feasibility testing and/or gauging organisational readiness for change (9.0%) [67, 116, 119, 124–127, 153, 171–173, 178, 182]. This often involved identifying factors that might help or hinder implementation via stakeholder interviews or focus groups [126, 127, 171, 178, 182]. Many of these efforts informed policies, guidelines and/or implementation plans [116, 124, 125, 172, 182].

Many of the 144 publications presented findings following the analysis of qualitative data to clarify stakeholder perceptions and attitudes re implementation, and/or the associated helpers and hindrances (54.2%) [43, 44, 46, 48, 49, 51, 52, 56–60, 62, 66, 68–90, 112, 114, 117–119, 122, 124–126, 128–130, 139–148, 154–156, 159–164, 166, 167, 170–172, 174–176, 180, 183]. These studies largely involved maternity care clinicians and pregnant women (84.6%). They also involved those with expertise in research and/or program implementation whose knowledge served to contextualise a framework [112], like frameworks that are internationally recognised [188], to optimise local relevance. Despite the value of some of these findings—like identifying factors that ‘helped’ or ‘hindered’ implementation, like knowledge training; service provider motivation; effective multilevel coordination; leadership; and effective communication [43, 46, 48, 49, 52, 56–58, 60, 62, 66, 68, 70–81, 83–92, 114, 117–119, 122–126, 128–130, 139–143, 145–148, 154, 156, 159–164, 166, 167, 170–172, 174, 175, 180, 183]—many of these publications noted methodological limitations, including a small sample, recall bias, self-report reliance and/or limited generalisability (74.3%).

### Theories, models and frameworks: present

Of the 14 publications that reported the use of a theory, model and/or framework, as per Nilsen’s [16] categories (and in order of most common), five referred to a determinant framework [189–193], four referred to an implementation theory [186, 194–196], two referred to a classic theory [197, 198], one referred to an evaluation framework [199] and one referred to a process model [200] (see Table 3). The remaining publication referred to the ‘evidence-based stages of implementation devised by the National Implementation Research Network (NIRN)’ [201], which appears to be beyond Nilsen’s categories—this might be because the framework is not espoused to be used in a linear fashion; but rather, its components are said to interact throughout the implementation process [202].

**Table 3:** Use of a theory, model and/or framework to guide implementation (*n* = 14)

Category	Publication	Nation	Aim	Design and/or method	Participants	Theory, model or framework	Use
Determinant frameworks	[192]	Morocco	‘understand the implementation process by identifying the characteristics of this intervention and the dimensions of the three systems which could act as barriers to/facilitators of the implementation process’	Case study (document analysis, focus groups, interviews, observation of educational sessions)	• Administrators (medical administration officers, administrative nurse cadres, health programmer), clinicians (consultant, midwives, nurses, obstetricians, physicians), managers (academic directors, medical directors nurse managers, midwifery managers and representatives), students, women (*n* = 107)	Consolidated framework for implementation research	Analyse qualitative data
	[191]	Australia	‘explore the enablers and barriers to implementation of the Australian smoking cessation in pregnancy guidelines’	Interviews	• Managers (obstetric, midwifery = 8), clinicians (midwives, obstetricians = 19; total = 27)	Theoretical domains framework	Identify implementation barriers
	[190]	Kenya	‘describes and analyses the implementation process, its strengths and challenges, and the lessons gained’	Mixed-methods (case narratives, document analysis, focus groups, interviews)	• Clinicians (community health workers, doctors, matrons, nurses), managers (district health program managers, coordinators), policymakers, professional association representatives (medical, nursing), women who delivered at the service in the last 6 months (interviews: *n* = 122) • Community leaders, community members, women who delivered at the service in the last 6 months (focus groups: *n* = 98) • Women who delivered at the service in the last 6 months (case narratives: *n* = 65)	Consolidated framework for implementation research	Analyse qualitative data
	[193]	Australia	‘describes the perceptions that midwives and nurses have about the BFHI [Baby Friendly Health Initiative] and examines factors that may facilitate or hinder the implementation process’	Focus groups	• Clinicians (child and family nurses, midwives, neonatal nurses), managers (clinical consultants, midwifery and child and family health nursing managers), student midwives (*n* = 132)	Diffusion of innovations model	Analyse qualitative data
	[189]	Australia	‘systematically assess evidence-practice gap in the multidisciplinary management of overweight and obesity… in pregnancy to inform an intervention to facilitate translating obesity guidelines into practice in a tertiary maternity service’	Survey	• Clinicians (dieticians, midwives, obstetricians, physiotherapists; *n* = 84)	Theoretical domains framework	Analyse qualitative data
Implementation theories	[195]	Australia	‘discuss how theory can be used to explore, understand and interpret implementation strategies and the impact of organisational context when evaluating new models of health service delivery’	Case studies	• RCT one: midwives (*n* = 8), women (*n* = 1000) • RCT two: midwives (*n* = 12), women (*n* = 2314)	Normalisation process model	Analyse qualitative data
	[186]	United Kingdom	‘develop an intervention to improve the quality and content of place of birth discussions between midwives and low-risk women and to evaluate this intervention in practice’	Mixed-methods (focus groups, interviews, questionnaires, midwife feedback visits, workshops)	• Stage 1: midwives (*n* = 38) • Stage 2: midwives (*n* = 58) • Stage 3: midwives (*n* = 66)	Capability, opportunity, motivation and behaviour (COM-B)	Guide intervention design
	[196]	United Kingdom	Gauge the ‘acceptability of the system changes to staff, as well as aids and hindrances to implementation and normalization of this complex intervention’	Process evaluation (interviews, observation)	• Maternity staff (*n* = 60), staff who deliver smoking cessation services (*n* = 39), staff of other organisations (*n* = 4; total = 103)	Normalisation process theory	Analyse qualitative data
	[194]	United Kingdom	‘explore the benefits, barriers and disadvantages of implementing an electronic record system (ERS). The extent that the system has become ‘normalised’ into routine practice was also explored’	Interviews	• Healthcare staff (doctors, healthcare assistants, midwives; total = 19)	Normalisation process theory	Analyse qualitative data
Classic theories	[198]	Spain	‘develop an instrument to measure variables that influence health care professionals’ behaviour with regard to the protection, promotion, and support of breastfeeding, especially one that related to the Baby-Friendly Hospital Initiative (BFHI), and to conduct a psychometric assessment’	Cross-sectional using a questionnaire	• Multidisciplinary working group that developed the questionnaire included (preventive medicine and public health physicians = 2; psychologists = 2; midwife=1; nurse = 1; paediatrician *n* = 1) • Expert groups that reviewed the questionnaire (clinicians=20; psychologists = 12; nurses = 6; paediatricians = 5; midwives = 3; general practitioners = 2) • Maternity and primary care clinicians who completed the questionnaire, including midwives, nurses, nursing assistants, physicians (*n* = 201)	Theory of reasoned action	Inform questionnaire development
	[197]	Australia	‘understand clinician factors that may influence the up- take, acceptance and use of the NLBB [Normal Labour and Birth Bundle]’	Mixed-methods (two focus groups, survey)	• Maternity care clinicians (midwives, consultant obstetricians, residents and registrars; *n* = 74)	Theory of planned behaviour	Analyse qualitative data
Evaluation framework	[199]	Zambia	‘explore perspectives, roles, achievements and challenges of the Safe Motherhood Action Groups (SMAG) programme in Kalomo, Zambia’	Interviews	• Action group members (*n* = 22), community leaders (*n* = 5), husbands (*n* = 3), manager (*n* = 1), mothers (*n* = 10), nurses (*n* = 5; total = 46)	PRECEDE-PROCEED	Analyse qualitative data
Process model	[200]	United States	‘set forth a new patient-centred implementation model informed by a qualitative study that explored women’s decisions, perceptions, and experiences of elective induction of labour’	Interviews	• Pregnant women (*n* = 29)	Ottawa model of research use (OMRU) framework	Analyse qualitative data
Additional framework	[201]	United Kingdom	Gauge the feasibility of implementing a maternity care intervention	Case study (pre-implementation survey, development and deployment of an implementation plan)	• Postnatal women (*n* = 250)	Stages of implementation framework	Describe and guide the translation of research into practice

Published within the last 10 years—between 2011 and 2019 (inclusive)—the 14 publications reported on studies conducted in hospital maternity units [189, 191, 194, 195, 197, 198, 200, 201] or across both hospital and community-based services [186, 190, 192, 196, 199]. These studies were conducted in Australia (35.7%) [189, 191, 193, 195, 197]; the UK (28.6%) [186, 194, 196, 201]; as well as the USA [200], Kenya [190], Morocco [192], Spain [198] and Zambia [199]. Collectively, this suggests the studies were chiefly conducted within western health systems. Several studies involved clinicians (57.1%) [186, 189, 191, 193, 194, 196–198], chiefly maternity care clinicians, like midwives [186, 189, 191, 193, 194, 196–198]. Others involved both maternity care clinicians and pregnant women [192, 195, 201]; only pregnant women [200]; or various participants including clinicians, policymakers, mothers, husbands and community leaders [190, 199]. This suggests that (prospective) recipients of care—be they women, their partners, their infants or their family members—were not always involved in these studies. Having described these 14 publications, the following sections explicate them with reference to Nilsen’s [16] categories.

### Determinant frameworks

Determinant frameworks ‘specify… types… classes or domains… of determinates and individual determinants, which act as barriers and enablers (independent variables) that influence implementation outcomes (dependent variables)’ [16]. Accordingly, they typically indicate an overarching aim to clarify what influences implementation and how. Only five publications referred to a framework that reflects this definition—notably, the consolidated framework for implementation research (CFIR) [190, 192], the theoretical domains framework (TDF) [189, 191] and a model to diffuse innovations [193].

To understand the characteristics of an intervention that influence its implementation, Abou-Malham and colleagues [192] used the CFIR with a conceptual model to elucidate role changes among clinicians [203]. Specifically, they used the CFIR to analyse (chiefly qualitative) data, collected via ‘focus groups… interviews… field notes, observation of educational sessions… and documents related to the implementation process’ [192]. The study involved 107 participants, most of who were midwifery educators (*n* = 29) and midwife practitioners (*n* = 17). The authors identified seven themes that helped implementation and 17 that hindered it, which collectively aligned with 22 of the 24 CFIR constructs. Following this, they suggested that when designing and implementing community-based interventions, it can be helpful to: use ‘knowledge transfer strategies such as interactive workshops’; use ‘collaborative’ and ‘participatory approach[es]’ to engage diverse stakeholders; enhance ‘communication mechanisms’; and improve ‘organisational readiness’ by increasing ‘financial, human and material resources’. Despite the potential value of these lessons, the authors acknowledged that their ‘case study’ might have limited relevance further afield, particularly given their use of ‘secondary sources of information’ and the limited resources and time for the study.

Warren and colleagues [190] also used the CFIR to understand what helped and hindered the implementation of an intervention across 13 Kenyan counties—namely, the respectful maternity care resource package—albeit retrospectively. They used this determinant framework because it emphasises stakeholder perceptions across all implementation phases, from its design to its associated outcomes. Although CFIR-use was previously limited to ‘disease-specific or targeted behaviour change interventions’, they used it iteratively to untangle some of the complexity associated with ‘policy, facility and community activities’. This involved using the CFIR to triangulate their analysis of qualitative data, including project documents, reports and interviews. Following this, the authors identified the characteristics associated with the intervention or individuals; the process domains; as well as the contextual factors that influenced implementation. Despite the value of the CFIR, some domains were described as ‘not contextually relevant’, because ‘this is one of the first studies to apply CFIR in sub-Saharan Africa’. As such, they ‘recommend[ed] further use and testing of the framework to different multifaceted interventions and health areas in the region’.

Schmied and colleagues [193] used a diffusion of innovations model to clarify the characteristics of an intervention that would influence its use. Accordingly, they concluded that intervention use was enhanced by its ‘relative advantage’, ‘compatibility with the midwifery philosophy of practice’ and ‘trialability’. Conversely, intervention use was compromised by the limited ‘observability of the benefits’, the ‘complexity’ of the intervention, its ‘inflexibility’ and limited ‘augmentation support’. According to the authors, although informative, the value of these lessons was hindered by methodological limitations, including limited participant involvement; participant bias, given their voluntary involvement; and the absence of other stakeholders who shape maternity care, notably non-nursing clinicians, women and their partners.

Unlike the aforesaid publications [190, 192], Longman and colleagues [191] used the TDF. The TDF categorises behaviour change into 14 domains, including knowledge, skills, intentions, goals, social influences and beliefs about capabilities. The authors used this taxonomy to ascertain the factors that would help and hinder the implementation of a maternity care intervention. Specifically, it informed their interviews with midwives, obstetricians and service managers regarding their experiences with the intervention to plan its implementation. The authors deemed the TDF comprehensive, ‘facilitating a thorough and systematic assessment of enablers and barriers’ to ultimately optimise the implementation of the intervention. In the absence of a critical appraisal or the expressed identification of methodological limitations, the authors did not acknowledge the shortcomings associated with the TDF.

Similarly, Wilkinson and Stapleton [189] used the TDF to clarify what enables obstetricians, midwives and allied health professionals to use obesity guidelines to manage overweight and obesity among pregnant women. Specifically, the TDF was used to inform the analysis of qualitative data. According to the authors, hindrances largely pertained to ‘Knowledge, Skills, Social and Professional Role/Identity, Beliefs about capabilities and Environmental context and resources’, while the helpers related to ‘Beliefs about consequences, Optimism and Social influences’. They recommended paying heed to these domains, to optimise guideline adherence and ultimately improve maternity care. Yet the potential value of these recommendations is curtailed by methodological limitations—notably, the ‘lower than desirable response rate’ and ‘disproportionate representation in some staff groups’.

### Implementation theories

As an explanatory proposition, implementation theories serve to understand how different practices are introduced, operationalised and sustained [16]. Only four publications referred to an implementation theory—these included the normalisation process theory [194–196]; and the capability, opportunity, motivation and behaviour (COM-B) [186]. The former suggests four determinants help to institutionalise different practices—namely, ‘coherence or sense making, cognitive participation or engagement, collective action and reflexive monitoring’ [16]. Although deemed to be an implementation theory, the normalisation process theory can also serve as an evaluation framework. Accordingly, by using the theory to inform the analysis of qualitative data, two publications reported on how it was used to evaluate interventions [195, 196]. The third described how the theory was used to determine the pros and cons of an electronic record; the factors that hindered its implementation; as well as the extent to which it had become routinised [194]. For comparative value, one of these publications reported on two case studies—‘one where a theoretical framework was used, the other where it was not’ [195]. According to the authors, the normalisation process theory ensured due recognition of the organisational context. It directed attention to ‘a new role for midwives and the support of key stakeholders in the organisation’ as well as the data required to understand how implementation might be optimised. Although the authors recognised that the retrospect use of theory limited their analysis to speculation, they indicated that the normalisation process theory enabled them to identify ‘the factors to be taken into account when planning and implementing complex interventions’.

Henshall and colleagues [186] used the COM-B to inform an intervention to improve and evaluate ‘the quality and content of place of birth discussions between midwives and low-risk women’. Specifically, they used it to categorise qualitative data, sourced via midwife interviews, to clarify their capability, opportunity and motivation, with reference to the intervention. This helped them to identify ‘intervention functions and potential behaviour change techniques’ to optimise its use, as evidenced by the evaluation. For instance, the authors averred that the ‘co-production’ of an ‘intervention package’ between ‘researches, women and midwives’, ‘substantially improved’ midwife ‘knowledge and confidence regarding place of birth’; specifically, the intervention ‘promot[ed]… discussions and aid[ed]… communication about place of birth options’—yet robust evidence to support these claims was limited, potentially compromised by the sample, which ‘may not be representative’ of all midwives and women.

### Classic theories

Classic theories are those sourced from disciplines beyond implementation science, including (but not limited to) ‘psychology, sociology and organisational theory, to… understand… aspects of implementation’ [16]. Only two publications referred such a theory—these included the theory of reasoned action [198]; and the theory of planned behaviour [197]. The former postulates that ‘behavioural intentions, which are the immediate antecedents to behaviour, are a function of salient information or beliefs about the likelihood that… a particular behaviour will lead to a specific outcome’ [204]. Extending this theory, the latter recognises the role of personal beliefs, whereby ‘The more resources and opportunities individuals think they possess, the greater should be their perceived behavioural control over behaviour’.

Bermejo and colleagues [198] used the theory of reasoned action to develop a questionnaire for nursing assistants, nurses, midwives and physicians re professional breastfeeding support. Reflecting the theory, the questionnaire gauged ‘beliefs, attitudes, subjective norms, and behavioural intention’. According to the authors, these domains helped to ensure due recognition of the personal and social dimensions of change, including ‘specific training’ needs, staff motivation and ‘interest’, ‘support from colleagues’ and staff ‘appraisal’ of workplace policies related to the intervention, all of which improved intervention use. Although informative, the authors acknowledged that sample bias limited their strength of their study.

Conversely, Wong Shee and colleagues [197] used the theory of planned behaviour to clarify why clinicians (do not) comply with evidence-based guidelines, with particular reference to ‘attitudes, subjective norms and perceived behavioural control’. This was achieved by using the theory to inform the collection and analysis of data, collected from midwives, obstetricians, general practice obstetricians, obstetric registrars and resident medical officers, via surveys and focus groups. Following this, the authors discovered that an intention to use the intervention was chiefly predicted by self-efficacy, positive social pressure and positive attitude. Furthermore, the theory directed their scholarly gaze to context, whereby intervention use was influenced by the regional location of the service. However, whether any of these perceptions actually influenced clinician use of the intervention was beyond the scope this publication.

### Evaluation frameworks

Sialubanje and colleagues [199] used an evaluation framework—namely, PRECEDE-PROCEED—to clarify ‘aspects of… implementation success’ [16]. Specifically, they used the framework to understand the perceived effectiveness of an intervention to increase the use of maternity services. This involved developing an interview schedule, guided by ‘the PRECEDE part in [the]… model, which prescribes consideration of health-related behavioural determinants and environmental conditions at multiple levels’ [199]; and analysing qualitative data, collected from diverse stakeholders—including mothers, husbands, volunteers and headmen (or village chiefs)—with reference to four a priori themes. However, the rationale for the sole focus on PRECEDE is not readily apparent, nor is the connection between PRECEDE-PROCEED and the four themes.

### Process models

Moore and colleagues [200] used a process model—namely, the Ottawa model of research use (OMRU)—to specify the ‘steps… [when] translating research into practice’ [16]—in this case, methods to optimise patient engagement in evidence-based care. Given the study focus, they modified the OMRU by incorporating the concepts of decision-making and patient activation [205]—that is, ‘[an] ability or [a] readiness… to engage in health behaviours that will maintain or improve… health status’ [200]. Following this, they mapped the findings from an exploratory study on the induction of labour, to the modified model to ‘verify implementation concepts and to identify potential gap’ [200]. According to the authors, the modified OMRU helped to recognise women as users of evidence—however, it had a limited capacity to adequately capture complex decision-making processes from women’s perspectives. This was the primary impetus for their new model—namely, the evidence-informed decision-making through engagement model.

### Additional framework

Cooper and Cameron [201] used the stages of implementation framework, devised by the national implementation research network, to translate evidence borne from research into practice. Although Nilsen [16] did not explicitly refer to these stages, it reflects an implementation science framework. This is because the authors used the stages of exploration, installation, as well as initial and full implementation to guide the staged introduction and use of an intervention. According to Cooper and Cameron, these stages enabled them to forecast what might hinder implementation and plan for these, accordingly. For instance, during the exploration stage, they identified a ‘local unmet need’ that served to build a case for a different device, thereby ‘gaining funding and support’ [201]. They were then able to prepare for the ‘installation phase’ by addressing the ‘practical factors that needed to be in place prior to formal service introduction’—these included securing the relevant equipment; as well as offering staff training and education. Furthermore, pilot-testing the device during the ‘initial implementation’ stage enabled the authors to ‘install… [it] more quickly and… fores[ee]… potential obstacles’ associated with its use, further afield—and soliciting ‘testimonies from patients and staff’ during the ‘full implementation’ stage helped to ‘overcom[e]… perceived barriers and gain… wider support’. Nevertheless, in the absence of a critical appraisal of the stages of implementation framework or the expressed identification of methodological limitations, the authors did not acknowledge the shortcomings associated with this framework.

## Discussion

Aligning healthcare with evidence-based (or -informed) practice can be challenging [5]. What clinicians do, how they do it, when they do it and who (or what) they do it with is shaped by myriad factors and processes, some of which are not readily conducive to change, like institutional logics [4, 6, 7, 11, 13]. Despite progress to address the oft-cited ‘quality chasm’ [206], evidence-based population health is far from ideal, particularly in low- and middle-income nations.

To advance evidence-based population health in an informed way, this article presented a scoping review to map and clarify implementation science in the seminal field of maternity care. This served to identify three key findings. First, most of the publications reported on studies regarding the factors that enabled implementation—such as knowledge; training; service provider motivation; effective multilevel coordination; leadership; and/or effective communication, with very limited expressed use of a theory, model or framework to inform implementation science. Second, when used, there was reference to: two theories (implementation theories = 4; classic theories = 2; total publications = 6); two frameworks (determinant frameworks = 5; evaluation framework = 1; total publications = 6); and one process model (total publications = 1). Third, when a theory, model or framework was used, it typically guided data analysis or, to a lesser extent, the development of data collection tools—rather than for instance, the design of the study.

Despite the value of the aforesaid findings, two methodological limitations warrant mention. First, the search strategy might have failed to identify relevant publications. In addition to the use of a focused search strategy, this is because of the myriad euphemisms for implementation and maternity. Second, given the varied understandings of implementation science, maternity care, theory, model and framework, it was not possible to verify the reported descriptions of these terms, as used by the authors.

Despite the aforesaid limitations, this scoping review suggests that implementation science in maternity care is largely limited to the study of helpers and hindrances, with little use of a theory, model or framework to inform implementation science. This finding has considerable implications for practitioners—including policymakers, health service managers and clinicians—as well as scholars.

For practitioners, this study highlights a range of factors that support evidence-based maternity care. These include knowledge, training, service provider motivation, effective multilevel coordination, leadership and/or effective communication. Given the findings of this scoping review, understanding and addressing these influences has the potential to improve the systematic uptake of evidence-based interventions in maternity care, to ultimately enhance ‘the health of women during pregnancy, childbirth and the postnatal period’ [30].

For scholars, there is the customary call for more research—however, what is specifically required is greater expressed use of diverse theories, models and frameworks. These might include organisational theories, like agency, situated change and/or institutional theories to guide which data are collected, as well as how they are collected, analysed, interpreted and used [207]. For instance, the use of institutional theory would direct scholarly attention to an organisation’s ‘rules of thumb’ [208]—its rules, requirements, customs and conventions. Yet, the judicious use of a theory, model or framework is equally important—for instance, it would be helpful to clarify why a theory, model or framework was (not) used, and the associated implications. To improve implementation science in maternity care, there is also considerable opportunity to strengthen the ways that theories, models and frameworks are used—these might include the design of a longitudinal, multi-site study to determine whether perceived helpers and hindrances actually influence the implementation of an intervention within different contexts. Although one publication included in this review intended to be longitudinal in design, the study was modified ‘Due to delay[ed]… introduce[tion]’ of the intervention [196]. Furthermore, given the relative absence of their voices within extant research, there is considerable opportunity to involve women, their partners and their family members in implementation science. Although they might not always be responsible for enacting evidence-based practices, their expertise is likely to ensure nuanced variation in maternity care, particularly that which is woman-focused and encourages personal agency to be exercised, as per international recommendations [31, 209–211]. Collectively, these research opportunities can advance maternity care by clarifying how and why evidence-based interventions in maternity care can be introduced and sustained; and distilling lessons that ‘hold… transferable applications to other settings, context, populations and possibly time periods’ [212].

## Conclusions

Aligning healthcare with evidence-based practice can be challenging, largely because what clinicians do, how they do it, when they do it and who they do it with is shaped by myriad factors and processes. Given that models and frameworks can help to describe phenomenon, and theories can help to both describe and explain it, evidence-based maternity care might be promoted via the greater expressed use of these to ultimately inform implementation science. This is particularly because only 14 of the 158 publications included in this review reported the use of a theory, model and/or framework—and of these, only 6 explicitly referred to a theory. There is clearly much opportunity to better inform the systematic uptake of evidence-based interventions in maternity care.

## Supplementary Information

**Additional file 1.**

## Data Availability

Not applicable.
